# Exploratory Analysis of Type 2 Diabetes Mellitus Metabolic Phenotypes and Their Association With Vitamin D Status in Primary Care Patients

**DOI:** 10.1111/dom.71275

**Published:** 2026-08-27

**Authors:** Liliane Viana Pires, Matheus Menezes‐Santos, Andréa Costa Goes, Heitor Siqueira Ribeiro, Luana Ferreira Alves, Beatriz Cruz dos Santos, Jéssyca Teles Barreto, Roberta Cristina Vieira Meneses, Ana Mara de Oliveira e Silva, Ana Hermínia Andrade e Silva, Sadraque Eneas de Figueiredo Lucena

**Affiliations:** ^1^ Nutritional Biochemistry Laboratory, Department of Nutrition, Center for Biological and Health Sciences Federal University of Sergipe São Cristóvão Brazil; ^2^ Nutrition Sciences Post‐Graduation Program, Department of Nutrition, Center for Biological and Health Sciences Federal University of Sergipe São Cristóvão Brazil; ^3^ Health Sciences Post‐Graduation Program, Center for Biological and Health Sciences Federal University of Sergipe São Cristóvão Brazil; ^4^ Management Innovation Institute, Cidade Ocidental Campus, Federal University of Goiás Cidade Ocidental Brazil; ^5^ Health Sciences Post‐Graduation Program, Faculty of Health Sciences University of Brasília Brasília Brazil; ^6^ Department of Statistics, Center for Exact and Natural Sciences Federal University of Paraíba João Pessoa Brazil; ^7^ Department of Statistics and Actuarial Sciences, Center for Exact and Technological Sciences Federal University of Sergipe São Cristóvão Brazil

**Keywords:** insulin resistance, observational study, primary care, real‐world evidence, type 2 diabetes

## Abstract

**Aims:**

This study aimed to perform an exploratory characterisation of metabolic phenotypes in primary care patients with type 2 diabetes mellitus (T2DM), using a predefined approach and examine their association with vitamin D status.

**Materials and Methods:**

This cross‐sectional study included 178 participants randomly selected in Sergipe, Brazil. Phenotypic clusters were derived using body mass index (BMI), glycated haemoglobin levels, age at diagnosis, insulin resistance (homeostatic model assessment (HOMA) for insulin resistance) and β‐cell function (HOMA‐B). To explore differences across phenotypes, we evaluated high‐sensitivity C‐reactive protein [hs‐CRP] and serum ferritin levels, waist circumference and BMI, serum concentrations of 25‐hydroxyvitamin D [25(OH)D] and therapeutic profile. Clustering was performed using the K‐medoids algorithm. Group comparisons were performed using the Kruskal–Wallis and Fisher's exact tests and associations were assessed using multivariable logistic regression. A *p*‐value of < 0.05 was considered significant.

**Results:**

Four metabolic phenotypes were identified: two more severe clusters (severe insulin‐deficient diabetes (SIDD)‐like and severe insulin‐resistant diabetes‐like) and two milder clusters (mild obesity‐related diabetes (MOD)‐like and mild age‐related diabetes (MARD)‐like). Significant differences were observed in age, insulin use, waist circumference and fasting glucose, triglycerides and hs‐CRP levels. Median 25(OH)D levels were lower in severe phenotypes compared with milder phenotypes. The SIDD‐like group, characterised by marked insulin deficiency, showed 3.68‐fold higher odds of vitamin D insufficiency (< 30 ng/mL) compared with the MARD‐like phenotype (*p* = 0.013).

**Conclusions:**

The metabolic phenotypes were explored in a primary care T2DM cohort, supporting their reproducibility across clinical settings. Vitamin D status varied by phenotype severity, with more severe phenotypes more likely to exhibit insufficiency.

## 1 Introduction

Type 2 diabetes mellitus (T2DM) is a complex, chronic non‐communicable disease with increasing global prevalence [1], characterised by diverse clinical and metabolic profiles [2]. Subgrouping approaches have been proposed to capture disease heterogeneity through distinct metabolic phenotypes. These phenotypes may differ in their responses to clinical and nutritional interventions due to underlying pathophysiological variations [3, 4].

Statistically derived metabolic phenotypes of T2DM are used to classify individuals into subgroups with distinct clinical features and may support clinical management by predicting phenotype‐specific outcomes [5, 6]. These phenotypes are typically defined using clinical, metabolic and biochemical variables, including glutamic acid decarboxylase autoantibodies (anti‐GAD), age at diagnosis, body mass index (BMI), glycated haemoglobin (HbA1c) levels and homeostasis model assessment (HOMA‐2) estimates of β‐cell function and insulin resistance [7].

Cardiovascular risk‐related phenotypes have also been described, including metabolically healthy normal weight, metabolically unhealthy normal weight, metabolically healthy obesity and metabolically unhealthy obesity, without relying solely on BMI as an isolated indicator [8]. Across these phenotypes, excess adiposity, low‐grade inflammation and increased oxidative stress are associated with reduced circulating levels of micronutrients essential for metabolic regulation [9, 10, 11].

Among these, vitamin D is notable for its genomic and non‐genomic roles in metabolic regulation [12]. It has been consistently associated with insulin secretion and β‐cell function through the binding of its active form, 1,25‐dihydroxyvitamin D [1,25(OH)_2_D_3_], to the vitamin D receptor. Additionally, the expression of 1α‐hydroxylase (CYP27B1) and vitamin D response elements in β‐cells promotes insulin gene transcription, proinsulin processing and insulin secretion [13, 14].

Vitamin D also modulates the expression of insulin receptors and glucose transporters in muscle, adipose tissue and liver, thereby enhancing cellular glucose uptake and reducing insulin resistance [15]. These effects contribute to the reduction of inflammatory markers and oxidative stress, which are implicated in insulin resistance and complications arising from poor metabolic control [13]. Moreover, substantial evidence links vitamin D deficiency with excess body weight across diverse populations [16, 17] as well as with poor glycemic control [18].

In this context, exploring differences across previously established T2DM metabolic phenotypes within a Primary Health Care (PHC) setting—particularly in publicly funded health services—and examining their association with vitamin D status are essential to address critical gaps in understanding disease heterogeneity and its clinical implications, including the role of nutrients in disease management. Furthermore, multidimensional phenotype‐based approaches that integrate clinical severity with nutritional and endocrine status, such as vitamin D, remain underexplored, despite their potential to guide tailored therapeutic strategies. Thus, this study aimed to perform an exploratory characterisation of metabolic phenotypes, using a predefined approach and examine their association with vitamin D status in individuals with T2DM receiving primary care within the Unified Health System.

## 2 Materials and Methods

### 2.1 Study Design and Population

This cross‐sectional analytical study was conducted in Sergipe, Brazil (latitude ~10° S), a tropical region with high solar radiation throughout all seasons. Data collection took place year‐round among 178 patients with physician‐diagnosed T2DM, defined according to the criteria of the Brazilian Diabetes Society [19]. Participants were recruited from 15 PHC units across three health macro‐regions of the state of Sergipe (Aracaju, Lagarto and Itabaiana) and were selected through simple random sampling from a list generated using Randomise software.

Individuals of both sexes, aged 20–70 years, receiving regular follow‐up in PHC were included in the analysis. Meanwhile, pregnant women and individuals with conditions that could interfere with inflammatory or metabolic profiles, including autoimmune diseases (e.g., systemic lupus erythematosus) and human immunodeficiency virus, acute inflammatory conditions or regular use of anti‐inflammatory medications and vitamin or mineral supplements, were excluded from the study.

The present study is a secondary exploratory analysis derived from baseline data of a parent clinical trial, whose sample size was calculated a priori. The original trial required a minimum of 95 participants (114 after allowing for 20% attrition), based on detecting a 1.0% difference in HbA1c with 90% power and *α* = 0.01. The present analysis included 178 participants.

The study was conducted in accordance with the Declaration of Helsinki and approved by the Research Ethics Committee of the Federal University of Sergipe (approval no. 5.173.073). All participants provided written informed consent.

### 2.2 Clustering Variables

For the identification of phenotypic clusters, five key variables were selected to emulate the pathophysiological taxonomy proposed by Ahlqvist et al. [7]. The anthropometric and glycemic control variables included (1) BMI (kg/m^2^), calculated from measured body weight and height and classified according to the World Health Organization [20] criteria for adults and Lipchitz [21] criteria for older adults (≥ 60 years); (2) HbA1c, measured by high‐performance liquid chromatography and expressed as a percentage (%); and (3) age at diagnosis (years), defined as the difference between the patient's chronological age at the time of assessment and the self‐reported duration of diagnosis. Proxies of glycemic homeostasis included (4) insulin resistance (HOMA‐IR) and (5) β‐cell function (HOMA‐B). Both indices were calculated using the HOMA model [22], available via the Oxford Calculator (version 2.2.3) (Diabetes Trials Unit, University of Oxford, Oxford, UK, https://www.rdm.ox.ac.uk/about/our‐clinical‐facilities‐and‐units/DTU/software/homa), based on fasting glucose and C‐peptide levels measured using enzymatic and electrochemiluminescence methods, respectively.

### 2.3 Characterisation Variables

A set of biochemical, anthropometric and clinical variables not included in the clustering step was selected for phenotype characterisation.

Inflammatory markers included high‐sensitivity C‐reactive protein (hs‐CRP), measured by immunoturbidimetry and serum ferritin, determined by chemiluminescence. Fasting glucose and total cholesterol levels were measured using enzymatic methods, whereas triglyceride and high‐density lipoprotein cholesterol (HDL‐c) levels were determined using colorimetric assays. Central adiposity was assessed by measuring waist circumference (WC) in accordance with the World Health Organization protocol [23]. Visceral fat percentage was estimated using bioelectrical impedance analysis (OMRON, model HBF‐514).

Serum 25‐hydroxyvitamin D [25(OH)D] concentrations were measured using immunoturbidimetry. Vitamin D status was classified as deficient (< 20 ng/mL), insufficient (20–29 ng/mL) or sufficient (≥ 30 ng/mL) [24]. The therapeutic profile data, including insulin use and oral antidiabetic agents biguanides, sulfonylureas, dipeptidyl peptidase‐4 (DPP‐4) inhibitors, sodium‐glucose transport 2 (SGLT2) inhibitors and thiazolidinediones (yes/no), were obtained from self‐reported clinical history using a structured questionnaire.

### 2.4 Statistical Analysis

The initial step involved data preparation for cluster analysis. The dataset was examined for missing values in the five input variables (age at diagnosis, BMI, HbA1c, HOMA‐B and HOMA‐IR). The initial sample comprised 179 patients; however, one missing value (0.56%) was identified in the age at diagnosis variable. This observation was excluded to preserve analytical consistency and avoid potential bias arising from imputation in a small dataset, yielding a final analytical sample of 178 individuals. All continuous variables were subsequently standardised using *z*‐scores (mean = 0 and standard deviation = 1) to ensure comparability across scales and facilitate appropriate application of the clustering algorithm.

For the identification of phenotypic subgroups, the K‐medoids algorithm, also known as partitioning around medoids (PAM) [25], was employed. This method was selected over centroid‐based algorithms (such as K‐means) due to its greater robustness to outliers, a particularly relevant characteristic when analysing asymmetrically distributed variables, including HOMA indices used in this study. The number of clusters (*k*) was predefined as 4 because the primary objective was to explore the metabolic subgroups originally proposed by Ahlqvist et al. [7], rather than to identify a new clustering structure. Accordingly, the clustering analysis was conducted as a replication of the original classification framework, consistent with previous exploratory or external validation studies of the Ahlqvist taxonomy [26, 27, 28]. To enhance methodological transparency, additional internal validation analyses were performed as sensitivity analyses, including the Average Silhouette Width, Gap Statistic, Davies–Bouldin Index, Calinski–Harabasz Index and bootstrap‐based Jaccard similarity coefficients. Because these indices supported different clustering solutions, they were interpreted as complementary assessments and did not alter the prespecified clustering strategy. The absence of autoimmunity markers (e.g., anti‐GAD antibodies) precluded the identification of the fifth autoimmune cluster described in the original taxonomy; therefore, the replication was restricted to the four non‐autoimmune subgroups: severe insulin‐deficient diabetes (SIDD), severe insulin‐resistant diabetes (SIRD), mild obesity‐related diabetes (MOD) and mild age‐related diabetes (MARD).

Euclidean distance was used as the dissimilarity metric. To ensure the robustness of the final solution and minimise convergence to local minima, the algorithm was run with multiple initial partitions (1000 random initialisations) was set prior to execution to ensure full computational reproducibility.

After assigning individuals to the four clusters using the PAM algorithm, a profiling analysis was conducted to interpret and label each subgroup. For this purpose, the medians and interquartile ranges (IQRs) of the five original (non‐standardised) classification variables were calculated for each cluster. The resulting profiles were then systematically compared with the pathophysiological taxonomy proposed by Ahlqvist et al. [7].

The labels ‘SIDD‐like’, ‘SIRD‐like’, ‘MOD‐like’ and ‘MARD‐like’ were assigned to each cluster based on their similarity to the definitions in the original study. In addition, the Kruskal‐Wallis test was used to confirm significant differences in the five input variables across the four groups, supporting their separation into distinct phenotypic clusters.

Finally, a characterisation analysis was conducted. In this step, the four phenotypes (SIDD‐like, SIRD‐like, MOD‐like and MARD‐like) were treated as the categorical independent variables. Continuous and ordinal variables were compared across the groups using the nonparametric Kruskal–Wallis test. When overall differences were significant, Dunn's post hoc tests with Holm adjustment for multiple comparisons were applied to identify pairwise group differences.

For categorical (nominal) descriptive variables, Fisher's exact test was applied as the global test. This approach was preferred over the chi‐square test due to low expected cell counts (i.e., *n* < 5) in some contingency table cells, which may compromise the validity of the chi‐square approximation [29]. Post hoc pairwise comparisons were subsequently performed using tests of proportions, with *p*‐values adjusted using the Holm–Bonferroni method. The level of statistical significance was set at *p* < 0.05.

Vitamin D status was categorised for the analysis of its association with the identified phenotypes. A cutoff value of < 30 ng/mL was used to define vitamin D deficiency or insufficiency, in accordance with the Endocrine Society guidelines [24]. The association was evaluated using multivariable binary logistic regression, with a minimally adjusted model including sex, age and educational level. Odds ratios (ORs) and their corresponding 95% confidence intervals (95% CIs) were reported. Variables identified in the literature as potential confounders were already incorporated into the construction of the phenotypes used as the exposure variable; therefore, further adjustment for these variables was not performed, as it could result in overadjustment bias.

All statistical analyses were conducted using R (version 4.5.1; R Core Team, 2025), in conjunction with the *cluster* package [30] for K‐medoids analysis, *dunn. test* [31] for post hoc comparisons and tidyverse [32] for data management and visualisation.

## 3 Results

### 3.1 Sample Characterisation and Metabolic Phenotype Identification

A total of 178 individuals with T2DM were included (median age: 58 years [IQR: 51.0–63.0]). Of these, 76 (42.7%) were older adults (≥ 60 years), whereas 91 (51.1%) were women. The median duration of T2DM was 6.0 years (IQR: 3.0–11.5). The prevalence of overweight and obesity among adults were 39.2% and 46.1%, respectively. In older adults, the prevalence of excess weight was 52.6%. Concerning glycemic control, the median values were 7.1% (IQR: 6.2–8.7) for HbA1c, 135.0 mg/dL (IQR: 103.0–190.8) for fasting plasma glucose, 2.4 (IQR: 1.7–3.4) for HOMA‐IR and 81.7 (IQR: 38.1–114.0) for HOMA‐B (data not shown in the table).

Four significant phenotypic subgroups were identified among individuals with T2DM receiving PHC (*n* = 178) (Table 1). Significant between‐group differences were observed across all five input variables included in the clusters (*p* < 0.001) (Table 1). The distribution of these variables across clusters is presented in Figure S1.

**TABLE 1:** Profiling of clustering variables according to metabolic phenotypes (*n* = 178).

Characteristic	SIDD‐like (*n* = 45)	SIRD‐like (*n* = 35)	MOD‐like (*n* = 48)	MARD‐like (*n* = 50)	*p*‐value*
Prevalence	25.1%	19.6%	26.8%	28.5%	
Age at diagnosis (years)	**42.0** ^a^ **[37.0**–**45.0]**	50.0^b^ [43.9–56.0]	45.0^a^ [38.8–52.2]	**57.5** ^c^ **[54.2**–**60.0]**	< 0.001
BMI (kg/m^2^)	29.6^a^ [26.7–31.5]	**33.4** ^b^ **[31.5**–**35.5]**	**26.4** ^*c*^ **[24.8**–**27.8]**	31.6^d^ [29.1–35.4]	< 0.001
HbA1c (%)	**10.1** ^a^ **[8.9**–**10.8]**	**6.0** ^b^ **[5.7**–**6.3]**	6.3^b^ [5.9–6.9]	7.5^c^ [6.5–8.1]	< 0.001
HOMA‐B	**30.0** ^a^ **[20.0**–**50.4]**	**145.9** ^b^ **[123.8**–**187.2]**	96.2^c^ [73.9–111.3]	70.5^d^ [45.9–92.0]	< 0.001
HOMA‐IR	**3.2** ^a^ **[2.1**–**4.6]**	**2.8** ^a^ [2.4–3.6]	**1.7** ^b^ **[1.4**–**2.2]**	2.3^c^ [1.6–3.1]	< 0.001

*Note:* Data are presented as median [interquartile range]. Bold values indicate the defining characteristics of each cluster.

Abbreviations: BMI, body mass index; HbA1c, glycated haemoglobin; HOMA‐B, proxy for β‐cell function; HOMA‐IR, proxy for insulin resistance; MARD, mild age‐related diabetes; MOD, mild obesity‐related diabetes; SIDD, severe insulin‐deficient diabetes; SIRD, severe insulin‐resistant diabetes.

* Global *p*‐values were obtained using the Kruskal–Wallis test. When appropriate, post hoc pairwise comparisons were performed using Dunn's test with Holm adjustment for multiple testing. Values sharing at least one superscript letter are not significantly different, whereas values with no superscript letters in common differ significantly (*p* < 0.05).

Internal validation metrics yielded different recommendations regarding the number of clusters, with no single criterion consistently supporting the same clustering solution. Bootstrap‐based Jaccard coefficients likewise indicated varying levels of cluster stability across the evaluated values of k. Because the objective of the present study was to replicate the Ahlqvist [7] classification rather than to identify a novel clustering structure, these analyses were interpreted as sensitivity analyses and did not alter the prespecified four‐cluster solution. Detailed results are provided in Table S1 and Figure S2.

The relative distributions of the clustering variables largely reproduced the principal phenotypic signatures originally described by Ahlqvist et al. [7] (Table S2). The SIDD‐like and MARD‐like clusters most closely reproduced the original phenotypic profiles; the former was characterised by the youngest age at diagnosis, the highest HbA1c levels and the lowest HOMA‐B values, whereas the latter comprised the oldest participants with relatively modest metabolic disturbances. Likewise, the SIRD‐like and MOD‐like clusters retained their core phenotypic characteristics, with SIRD‐like exhibiting the highest BMI and elevated HOMA‐IR and MOD‐like showing the lowest HOMA‐IR. Some differences were observed, including elevated HOMA‐IR in both the SIDD‐like and SIRD‐like clusters. Overall, these findings support the phenotypic correspondence between the clusters identified in the present study and the original Ahlqvist et al. [7] taxonomy.

### 3.2 Clinical Characterisation and Exploratory Analysis of Metabolic Phenotypes

Additional clinical and laboratory characteristics not included in clustering are presented in Table 2. The most notable finding was the therapeutic profile (*p* < 0.001), which differed significantly across groups. Insulin use was highest in the SIDD‐like phenotype (37.8%). *Post hoc* analysis confirmed that this prevalence exceeded that of the SIRD‐like (2.9%; *p* = 0.004), MOD‐like (8.3%; *p* = 0.008) and MARD‐like (10.0%; *p* = 0.010) phenotypes. The SIRD‐like phenotype exhibited the lowest prevalence of insulin therapy. Vitamin D insufficiency/deficiency was more frequent in the SIDD‐like phenotype than in the other phenotypes.

**TABLE 2:** Demographic, clinical, anthropometric, and inflammatory characterisation of the metabolic phenotypes (*n* = 178).

Characteristic	SIDD‐like (*n* = 45)	SIRD‐like (*n* = 35)	MOD‐like (*n* = 48)	MARD‐like (*n* = 50)	*p*‐value*
Demographic profile					
Female (*n*, %)	16 (35.6%)	20 (57.1%)	25 (52.1%)	30 (60.0%)	0.091
Age (years)	52.0^a^ [46.0–56.0]	57.0^b^ [52.0–62.0]	56.5^ab^ [46.8–60.0]	64.0^c^ [61.0–67.0]	< 0.001
Clinical validation					
Use of insulin (*n*, %)^1^	17 (37.8%)^a^	1 (2.9%)^b^	4 (8.3%)^b^	5 (10.0%)^b^	< 0.001
Anthropometry and adiposity					
Waist circumference (cm)	103.4^a^ [95.5–110.8]	110.2^b^ [103.0–118.0]	95.4^c^ [88.6–99.4]	108.4^b^ [102.0–116.8]	< 0.001
Visceral fat (%)	11.0^a^ [8.0–16.0]	14.0^b^ [12.0–17.0]	10.0^c^ [7.2–12.0]	13.0^b^ [12.0–16.0]	< 0.001
Inflammation and metabolic risk					
hs‐CRP (mg/L)	3.2^a^ [2.1–10.3]	2.5^ab^ [1.8–4.9]	1.7^b^ [0.8–3.4]	3.0^a^ [1.6–9.8]	0.004
Ferritin (ng/mL)	175.0 [103.2–323.8]	117.2 [78.0–282.6]	125.0 [57.6–204.8]	157.4 [76.1–290.3]	0.123
Fasting serum glucose (mg/dL)	216.0^a^ [193.0–275.0]	101.0^b^ [91.5–108.5]	111.0^c^ [96.0–136.0]	144.5^d^ [121.5–173.0]	< 0.001
Triacylglycerol (mg/dL)	173.0^a^ [114.0–289.0]	147.0^ab^ [114.5–210.5]	133.5^b^ [90.0–180.2]	145.0^b^ [115.0–185.8]	0.037
Total cholesterol (mg/dL)	199.0 [162.0–235.0]	189.0 [164.0–207.0]	191.5 [169.0–215.2]	182.5 [164.2–208.0]	0.597
HDL‐c (mg/dL)	46.0 [38.0–51.0]	49.0 [43.5–57.0]	47.0 [42.8–55.2]	47.5 [42.2–54.0]	0.147
Nutricional					
25(OH)D (ng/mL)	27.5 [23.0–36.1]	31.9 [23.8–37.4]	33.8 [28.7–43.7]	31.9 [24.4–36.7]	0.066
Vitamin D insufficiency/deficiency, *n* (%)	28 (62.2%)^a^	15 (42.9%)^ab^	15 (31.2%)^b^	20 (40.0%)^ab^	0.022

*Note:* Data are presented as median [interquartile range] or *n* (%).

Abbreviations: 25(OH)D, 25‐hydroxyvitamin D; HDL‐c, high‐density lipoprotein cholesterol; hs‐CRP, C‐reactive protein; MARD, mild age‐related diabetes; MOD, mild obesity‐related diabetes; SIDD, severe insulin‐deficient diabetes; SIRD, severe insulin‐resistant diabetes.

^1^ *p*‐values calculated using Fisher’s exact test.

* Global *p*‐values were obtained using the Kruskal–Wallis test (continuous and ordinal variables) or Fisher's exact test (categorical variables). When appropriate, post hoc pairwise comparisons were performed using Dunn's test (continuous/ordinal variables) or pairwise comparisons of proportions (categorical variables), with Holm adjustment for multiple testing. Values sharing at least one superscript letter are not significantly different, whereas values with no superscript letters in common differ significantly (*p* < 0.05).

Demographic analysis demonstrated significant differences in age across clusters (*p* < 0.001). Consistent with its nomenclature (‘age‐related’), the MARD‐like phenotype (median: 64 years) was characterised by a significantly older age (*p* < 0.001). By contrast, the SIDD‐like cluster (median: 52 years) was the youngest, with a significantly lower age compared with both the SIRD‐like (median: 57 years, *p* = 0.011) and MARD‐like groups. Sex distribution did not differ significantly among the clusters. The SIDD‐like phenotype showed the lowest proportion of women (35.6%), whereas the MARD‐like (60.0%) and SIRD‐like (57.1%) phenotypes exhibited the highest female proportions.

The most pronounced differentiation was observed in central adiposity (*p* < 0.001). The MOD‐like phenotype presented significantly lower WC (median: 95.4 cm) and visceral fat (median: 10.0%) (*p* < 0.05 for all pairwise comparisons with the MOD‐like group, Dunn's test). The highest adiposity levels were observed in the SIRD‐like and MARD‐like clusters (median WC: 110.2 and 108.4 cm, respectively; median visceral fat percentages: 14.0% and 13.0%, respectively). These values were significantly higher than those observed in the SIDD‐like and MOD‐like clusters.

The phenotypes exhibited distinct inflammatory and metabolic profiles. The SIDD‐like phenotype exhibited higher fasting glucose levels compared with the other phenotypes (*p* = 0.011) and higher triacylglycerol levels compared with the MOD‐like phenotype (*p* = 0.011), resulting in significant overall between‐group differences (*p* < 0.001 and *p* = 0.037, respectively). No significant differences were observed between the groups for total cholesterol and HDL‐c levels (Table 1).

The hs‐CRP levels differed across groups (*p* = 0.004), with the MOD‐like phenotype showing lower levels (median: 1.7 mg/L) compared with the SIDD‐like (median: 3.2 mg/L; *p* = 0.002) and MARD‐like phenotypes (median: 3.0 mg/L; *p* = 0.018). The MOD‐like group also showed a non‐significant trend toward lower hs‐CRP levels than the SIRD‐like group (median hs‐CRP: 2.5 mg/L; *p* = 0.091). Ferritin levels did not differ among the groups (*p* = 0.123) and no significant pairwise differences were observed in post hoc analyses (*p* ≥ 0.066).

Serum 25(OH)D concentrations evaluated as continuous variables showed a non‐significant trend toward variation across the four phenotypes (*p* = 0.066; Table 2). However, in a secondary exploratory analysis, when phenotypes were grouped by clinical severity (combining SIDD‐like and SIRD‐like as severe phenotypes vs. MOD‐like and MARD‐like as milder phenotypes), individuals with severe phenotypes exhibited significantly lower median 25(OH)D levels compared to those with milder phenotypes (28.6 ng/mL [IQR: 23.4–36.8] vs. 32.4 ng/mL [IQR: 25.8–39.6], *p* = 0.027; Figure 1).

**FIGURE 1:**
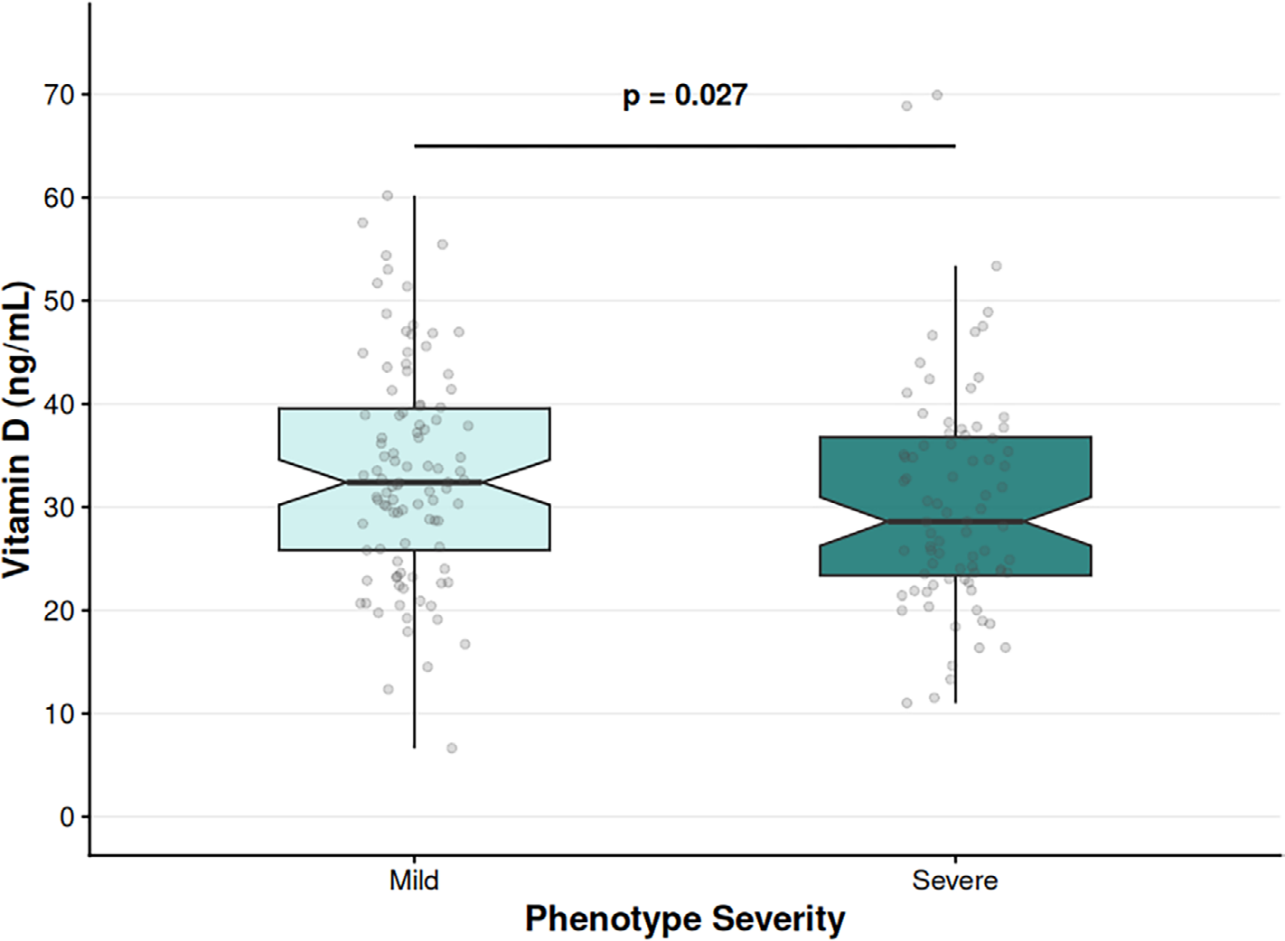
Serum 25(OH)D concentrations according to T2DM phenotypic severity. ‘Severe’ refers to SIDD‐like and SIRD‐like clusters; ‘Mild’ refers to MOD‐like and MARD‐like clusters.

Because vitamin D insufficiency represents a clinically relevant threshold, we subsequently evaluated the association between phenotypes and vitamin D status (< 30 ng/mL) using multivariable logistic regression (Table 3). In this adjusted analysis, individuals in the SIDD‐like phenotype had 3.68‐fold higher odds of vitamin D insufficiency compared with the MARD‐like reference group (95% CI: 1.34–10.56; *p* = 0.013). Moreover, female sex was independently associated with higher odds of vitamin D insufficiency (OR = 2.63, 95% CI: 1.36–5.24, *p* = 0.004), whereas age and educational level were not significantly associated (Table 3).

**TABLE 3:** Multivariable logistic regression analysis of vitamin D insufficiency (< 30 ng/mL) according to T2DM phenotypes.

Predictor variables	Vitamin D status^a^		
	Odds ratio (OR)	CI 95%	*p*‐value
Phenotypes (Ref: MARD‐like)			
SIDD‐like	3.68	1.34–10.56	0.013
SIRD‐like	1.21	0.46–3.20	0.692
MOD‐like	0.83	0.31–2.20	0.717
Adjustments			
Sex (female)	2.63	1.36–5.24	0.004
Age (years)	1.01	0.96–1.06	0.546
Education level (Ref: low)			
Medium (secondary education)	1.47	0.75–2.93	0.256
High (higher education)	2.42	0.71–9.02	0.163

Abbreviations: CI, confidence interval; MARD, mild age‐related diabetes; MOD, Mild obesity‐related diabetes; SIDD, severe insulin‐deficient diabetes; SIRD, severe insulin‐resistant diabetes.

^a^ Vitamin D status (reference = Adequate; dependent = Insufficiency < 30 ng/mL).

Finally, no significant associations were observed between metabolic phenotypes and the prevalence of clinical comorbidities or overall pharmacotherapy profiles. The prevalence of hypertension (*p* = 0.525), kidney disease (*p* = 0.421) and the use of oral antidiabetic agents (*p* = 0.510)—including biguanides (*p* = 0.954), DPP‐4 inhibitors (*p* = 0.239) and SGLT2 inhibitors (*p* = 0.811)—did not differ significantly across groups (Table S3).

## 4 Discussion

This study provides an exploratory analysis of the SIDD‐like, SIRD‐like, MOD‐like and MARD‐like phenotypes in individuals with T2DM receiving care in a PHC setting. Notably, the more severe phenotypes exhibited lower serum 25(OH)D concentrations compared with the milder phenotypes. Moreover, the SIDD‐like phenotype was associated with higher odds of vitamin D insufficiency relative to the MARD‐like reference group.

Different metabolic phenotypes have been described in individuals with T2DM, linking nutritional status to the presence or absence of metabolic alterations and highlighting differences in the clinical and metabolic profiles of this population [8]. Given the marked heterogeneity of the metabolic profile in T2DM and its implications for clinical management, phenotype‐based stratification incorporating disease‐related characteristics may support more precise therapeutic decision‐making [33, 34, 35], particularly in populations with diverse socioeconomic backgrounds receiving care in public health systems.

A German cohort study following individuals newly diagnosed with type 1 diabetes mellitus and T2DM over 5 years demonstrated substantial variability in clinical, metabolic and immunological profiles, along with dynamic transitions between phenotypes over time. These changes were associated with differential risks of complications and comorbidities [3]. Collectively, these findings highlight the importance of early identification of metabolic phenotypes to enable more targeted therapeutic strategies.

Insulin resistance represents a central pathophysiological mechanism in T2DM. It leads to chronic hyperglycemia that activates pro‐inflammatory pathways and amplifies metabolic stress, thereby perpetuating a cycle of low‐grade chronic inflammation [36]. The SIDD‐like phenotype exhibited the highest HbA1c levels, while both the SIDD‐like and SIRD‐like phenotypes presented higher insulin resistance compared to the milder phenotypes, corroborating the pathophysiological trajectory established in the literature [36]. Notably, individuals with the MOD‐like phenotype in our study exhibited overweight—rather than frank obesity—alongside normal‐to‐borderline insulin resistance. In the framework proposed by Ahlqvist et al. [7], this cluster was characterised by obesity without insulin resistance. Nevertheless, the label ‘MOD’ was retained to maintain consistency with the original nomenclature, despite the milder adiposity observed in our population.

In our study, markers of inflammation and metabolic risk were more pronounced in individuals with the SIDD‐like phenotype, reinforcing its more unfavourable metabolic profile, potentially driven by underlying inflammatory and oxidative stress pathways. In this context, inflammation and oxidative stress have been consistently linked to poorer metabolic control, particularly in insulin‐resistant states [37]. Furthermore, the reduced β‐cell function observed in this phenotype contributes to persistent hyperglycemia (as reflected by higher HbA1c levels), which in turn promotes glucotoxicity. This process further impairs β‐cell function, thereby sustaining inflammatory and oxidative stress pathways that underlie the poorer metabolic profile of this phenotype [38]. Besides, the insulin‐deficient profile exhibits an impaired capacity to suppress hepatic gluconeogenesis and adipose tissue lipolysis, thereby exacerbating hyperglycemia and elevating free fatty acid levels [38]. Conversely, the insulin‐resistant profile preserves compensatory hyperinsulinemia, allowing years of maintained glucose tolerance before β‐cell failure occurs [39].

Concerning the elevated triacylglycerol levels observed in the SIDD‐like phenotype in our study, the available evidence supports a link between lipid abnormalities and poorer glycemic control in T2DM [40, 41]. Excess circulating lipids may induce lipotoxicity in pancreatic β‐cells, resulting in apoptosis and impaired insulin synthesis and secretion. Consequently, these processes contribute to elevated blood glucose levels [42]. Additionally, this relationship is inherently bidirectional, as chronic hyperglycemia resulting from absolute or relative insulin deficiency and/or insulin resistance further elevates circulating triacylglycerol levels through enhanced lipolysis secondary to impaired insulin action [43]. These mechanisms align with the reduced β‐cell activity, higher HbA1c levels and elevated triacylglycerol levels observed in this phenotype.

The frequency of insulin use was significantly higher in the SIDD‐like phenotype in our study. This finding is consistent with the therapeutic requirements associated with its metabolic profile, characterised by endogenous insulin deficiency and poorer glycemic control. A previous study also reported that the SIDD phenotype exhibited the poorest glycemic control and the lowest β‐cell activity. However, insulin use (*n* = 1) was observed only in the SIRD phenotype, suggesting differences among the study populations in terms of disease severity and, consequently, in therapeutic needs, such as the use of exogenous insulin [44].

The SIRD‐like phenotype exhibited compensatory hyperinsulinemia, as reflected by higher HOMA‐B. This paradoxically translated into better glycemic control in this sample. A previous study also reported higher BMI, greater β‐cell activity and lower HbA1c among individuals with the SIRD phenotype [44]. However, this study also observed the highest HOMA‐IR in both the SIRD‐like and SIDD‐like phenotype. Collectively, these findings suggest shared clinical characteristics and a comparable pathophysiological trajectory between the studied populations.

Concerning the relationship between glycemic and lipid profile markers and vitamin D, individuals in the more severe phenotypes exhibited lower serum 25(OH)D levels. The prevalence of vitamin D deficiency and insufficiency among individuals with T2DM is high in many regions worldwide [45, 46]. Previous studies have consistently reported associations between vitamin D deficiency and classical T2DM‐related phenotypes [47]. These phenotypes include metabolic syndrome, obesity [48], hypertension, dyslipidemia, poorer glycemic control and micro‐ and macrovascular complications [49, 50]. However, phenotype‐based approaches that integrate multiple dimensions to predict both disease severity and vitamin D status remain underexplored.

The SIDD‐like and SIRD‐like phenotypes in our sample are characterised by increased insulin resistance, which represents a central feature of these subgroups. Evidence regarding the association between vitamin D deficiency or insufficiency and insulin resistance is consistent across the literature [51, 52]. Observational studies in populations with obesity, T2DM, metabolic syndrome and polycystic ovary syndrome have consistently demonstrated direct associations between vitamin D deficiency and insulin resistance [53, 54, 55]. The proposed mechanisms underlying this relationship involve several key pathways. These include reduced insulin receptor expression, increased systemic inflammation and alterations in intracellular insulin signalling [56]. Additionally, elevated parathyroid hormone levels may impair insulin‐stimulated glucose uptake [57].

As previously mentioned, the SIRD‐like phenotype exhibited the highest BMI among the groups. When grouped with the more severe phenotypes, it was characterised by lower serum 25(OH)D concentrations. Vitamin D deficiency and insufficiency are widely associated with overweight and obesity across diverse populations [16, 17]. The mechanisms underlying this association are thought to involve the sequestration of vitamin D in adipose tissue and volumetric dilution in individuals with excess body weight. Both processes reduce circulating vitamin D concentrations and may impair its biological functions, including its role in glucose metabolism [58, 59, 60].

Individuals classified as the SIDD‐like phenotype had higher odds of vitamin D insufficiency than those in the MARD‐like phenotype. This phenotype is considered the most metabolically severe because it is characterised by greater β‐cell dysfunction and poorer glycemic and metabolic control. However, the association between vitamin D concentrations and this phenotype is likely bidirectional. Most studies have investigated vitamin D status as a predictor of poor metabolic control in T2DM, suggesting that vitamin D deficiency may reduce vitamin D receptor (VDR) activation in pancreatic β‐cells, thereby impairing pathways involved in insulin secretion and contributing to progressive β‐cell dysfunction [61, 62, 63].

Overall, persistent hyperglycemia‐associated inflammation and oxidative stress impair vitamin D metabolism, reducing its bioavailability by suppressing key hydroxylation enzymes (hepatic CYP2R1, which converts cholecalciferol to 25(OH)D and renal CYP27B1, which produces active 1,25(OH)D) and enhancing catabolic CYP24A1 [64, 65]. Thus, reduced concentrations of 25(OH)D and/or 1,25(OH)D may impair VDR‐mediated genomic actions, particularly the regulation of genes involved in anti‐inflammatory responses, glucose metabolism and insulin homeostasis [63, 66]. Furthermore, identifying which T2DM subgroups harbour the highest risk of vitamin D deficiency/insufficiency allows for phenotype‐guided screening, helping clinicians to prioritise targeted assessment and tailored nutritional support for specific high‐risk phenotypes.

Additionally, female sex was associated with higher odds of vitamin D insufficiency [67, 68, 69]. Women generally have a higher body fat percentage and vitamin D may be sequestered in adipose tissue, reducing circulating levels [58, 59]. Other contributing factors include more frequent sunscreen use [70], pregnancy and lactation [71, 72] and the postmenopausal period [73].

Considering the growing interest in identifying metabolic phenotypes of T2DM for implementation in health care systems, data on these subgroups in PHC settings and their association with vitamin D status remain limited. Although individuals were evaluated at different treatment stages, which could potentially affect phenotype interpretability, it is noteworthy that key confounding variables, such as oral antidiabetic drug use and the presence of comorbidities, did not differ across phenotypes, thereby minimising the likelihood of misclassification. Beyond these aspects, evaluating patients across different treatment stages is essential for assessing the practical utility of clustering as a tool to guide personalised treatment. Even if a phenotype has been modified over time by disease trajectory or treatment, identifying the patient's current metabolic state remains clinically relevant for tailoring ongoing therapy.

In clinical practice, the phenotypic stratification enables risk‐stratified personalised management rather than a one‐size‐fits‐all treatment model. Identifying severe phenotypes (SIDD‐like and SIRD‐like) allows clinicians to prioritise high‐risk patients for intensive monitoring and early specialised referral, whereas milder phenotypes (MOD‐like and MARD‐like) can be managed with routine primary care follow‐up. Furthermore, this approach guides phenotype‐specific pharmacotherapy and lays the foundation for proxy‐based decision tools using routine clinical variables. Ultimately, it provides more precise care than isolated biomarker assessment while highlighting the importance of evaluating circulating vitamin D concentrations given its systemic metabolic role.

The strengths of this study include the exploratory replication of metabolic phenotypes using a robust statistical approach in a population from PHC settings. Additionally, the study integrated these phenotypes as exposure variables across nutritional and metabolic dimensions, which were reflected in the individual phenotype components. However, some limitations should be acknowledged. First, the absence of anti‐GAD antibody measurements precluded the identification of the fifth subgroup described by Ahlqvist et al. [7]. Second, the cross‐sectional design limits causal inference regarding the relationship between the identified phenotypes and vitamin D status, while the relatively small sample size may have reduced the statistical power to detect differences in key secondary outcomes, such as total cholesterol and HDL‐c levels. Third, individual sun exposure habits were not measured. However, this potential bias is attenuated by the fact that the study population resides in a tropical region with consistently high, year‐round solar irradiance (latitude ~10° S), which minimises seasonal fluctuations in 25(OH)D levels. Nevertheless, this setting may limit the generalizability of our findings to populations with different levels of sunlight exposure. Despite these limitations, the findings provide valuable insights into metabolic phenotypes among individuals with T2DM in PHC settings. They also have potential implications for informing treatment strategies and guiding referral for specialised care.

## 5 Conclusion

The exploratory analysis of metabolic phenotypes in individuals with T2DM in primary care revealed distinct clinical and metabolic characteristics. Individuals with severe phenotypes exhibited lower circulating 25(OH)D concentrations. In addition, the SIDD‐like phenotype was associated with higher odds of vitamin D insufficiency. However, these findings do not establish a causal relationship but highlight the potential role of vitamin D as an important metabolic regulator, extending beyond its classical function in bone homeostasis. Therefore, the identification of clinical and metabolic phenotypes in T2DM may support personalised management, although the application of complex statistical models in routine clinical practice remains a challenge.

## Author Contributions

L.V.P., M.M.‐S., B.C.S., L.F.A., J.T.B., R.C.V.M. were responsible for data collection. A.C.G., H.S.R. and A.M.O.S. contributed to the discussion of the study. L.V.P., M.M.‐S. and S.E.F.L. prepared the initial draft of the manuscript. All authors reviewed and critically revised the manuscript. S.E.F.L. and A.H.A.S. performed the statistical analysis.

## Funding

This study was funded by the National Research Council (CNPq) [CNPq/MS/SAPS/DEPROS no. 27/2020—process: 443162/2020‐4] and the Coordination for the Improvement of Higher Education Personnel (CAPES) [financial code 001]. M.M.‐S., B.C.S., L.F.A., J.T.B. and R.C.V.M. received scholarship grants from CAPES.

## Conflicts of Interest

The authors declare no conflicts of interest.

## Supporting information


**Figure S1:** Distribution of classification variables across the identified metabolic phenotypes (*n* = 178).
**Figure S2:** Evaluation of internal validation indices across alternative clustering solutions (*k* = 2 to *k* = 8). (A) Average Silhouette width (higher values indicate better‐defined clusters); (B) gap statistic (higher values indicate greater separation); (C) Davies–Bouldin index (lower values indicate greater cluster separation and compactness); (D) Calinski–Harabasz index (higher values indicate superior cluster separation).
**Table S1:** Internal validity and bootstrap stability metrics for clustering solutions ranging from *k* = 2 to *k* = 8.
**Table S2:** Comparison of phenotypic signatures between the original Ahlqvist [7] taxonomy and the present study.
**Table S3:** Clinical comorbidities, pharmacological therapy and social determinants across T2DM phenotypes (*n* = 177).

## Data Availability

The data that support the findings of this study are available from the corresponding author upon reasonable request.
