# Supplementary material for: Exploratory Analysis of Type 2 Diabetes Mellitus Metabolic Phenotypes and Their Association With Vitamin D Status in Primary Care Patients
Source: Diabetes Obes Metab. 2026 Aug 27;28(11):10603–14. doi: 10.1111/dom.71275 (PMC13646376; doi:10.1111/dom.71275)
Supplement: Supplementary file 1 [file DOM-28-10603-s001.docx]

Tables and Figures (Supplementary Material)

**Supplementary Figure 1.** Distribution of classification variables across the identified metabolic phenotypes (n = 178). Abbreviations: SIDD, Severe Insulin-Deficient Diabetes; SIRD, Severe Insulin-Resistant Diabetes; MOD, Mild Obesity-Related Diabetes; MARD, Mild Age-Related Diabetes; BMI, Body Mass Index; HbA1c, Glycated Hemoglobin; HOMA-B, proxy for β-cell function; HOMA-IR, proxy for insulin resistance.

**Supplementary Figure 2**. Evaluation of internal validation indices across alternative clustering solutions (k = 2 to k = 8). (A) Average Silhouette width (higher values indicate better-defined clusters); (B) Gap statistic (higher values indicate greater separation); (C) Davies–Bouldin index (lower values indicate greater cluster separation and compactness); (D) Calinski–Harabasz index (higher values indicate superior cluster separation).

**Supplementary Table 1.** Internal validity and bootstrap stability metrics for clustering solutions ranging from k = 2 to k = 8.

| **k*** | **Average Silhouette** | **Gap Statistic** | **Davies–Bouldin** | **Calinski–Harabasz** | **Mean Jaccard**** |
| --- | --- | --- | --- | --- | --- |
| 2 | 0.2382 | 0.5831 | 1.4617 | 57.7128 | 0.8114 |
| 3 | 0.1924 | 0.5838 | 1.5166 | 48.4347 | 0.6165 |
| 4 | 0.1851 | 0.5809 | 1.7198 | 45.349 | 0.5818 |
| 5 | 0.1937 | 0.5958 | 1.5927 | 45.8032 | 0.6279 |
| 6 | 0.1804 | 0.5901 | 1.7169 | 42.0844 | 0.5809 |
| 7 | 0.1675 | 0.5943 | 1.8434 | 40.4180 | 0.5330 |
| 8 | 0.1647 | 0.5955 | 1.8151 | 38.2183 | 0.5383 |

*Number of clusters. **Mean bootstrap Jaccard similarity coefficient.

Internal cluster validity was assessed using the Average Silhouette Width, Gap Statistic, Davies–Bouldin Index, and Calinski–Harabasz Index. Cluster stability was evaluated using bootstrap resampling (1,000 replications) and summarized by the mean Jaccard similarity coefficient across clusters. These analyses were performed as complementary sensitivity analyses and were not used to redefine the prespecified four-cluster solution.

**Supplementary Table 2.** Comparison of phenotypic signatures between the original Ahlqvist [7] taxonomy and the present study.

| Phenotype | Original description  (Ahlqvist et al.) | Present study |
| --- | --- | --- |
| SIDD-like | Early onset, poor glycaemic control, low BMI, and low β-cell function | Youngest age, highest HbA1c, and low β-cell function |
| SIRD-like | High insulin resistance and high BMI | Highest BMI, elevated HOMA-IR, and highest HOMA-B |
| MOD-like | Obesity without marked insulin resistance | Overweight without marked insulin resistance |
| MARD-like | Oldest age and modest metabolic disturbances | Oldest age, moderate HbA1c, and intermediate HOMA-B |

Abbreviations: SIDD, Severe Insulin-Deficient Diabetes; SIRD, Severe Insulin-Resistant Diabetes; MOD, Mild Obesity-Related Diabetes; MARD, Mild Age-Related Diabetes; BMI, Body Mass Index; HbA1c, Glycated Hemoglobin; HOMA-B, proxy for β-cell function; HOMA-IR, proxy for insulin resistance.

**Supplementary Table 3.** Clinical comorbidities, pharmacological therapy, and social determinants across T2DM phenotypes (n = 177).

| Characteristics | SIDD-like  (*n* = 45) | SIRD-like  (*n* = 35) | MOD-like  (*n* = 48) | MARD-like  (*n* = 50) | *p*-value^1^ |
| --- | --- | --- | --- | --- | --- |
| *Comorbidities* | | | | | |
| Hypertension | 31 (78.0%) | 25 (71.4%) | 31 (64.6%) | 39 (78.0%) | 0.525 |
| Kidney Disease | 0 (0.0%) | 0 (0.0%) | 2 (4.2%) | 2 (4.0%) | 0.421 |
| *Therapeutic Profile* | | | | | |
| Oral antidiabetic agents | 42 (93.3%) | 32 (91.4%) | 46 (95.8%) | 49 (98.0%) | 0.510 |
| Biguanides | 41 (91.1%) | 31 (88.6%) | 42 (87.5%) | 45 (90.0%) | 0.954 |
| Sulfonylureas | 21 (46.7%) | 7 (20.0%) | 21 (43.8%) | 17 (34.0%) | 0.064 |
| DPP-4 inhibitors | 3 (6.7%) | 0 (0.0%) | 5 (10.4%) | 2 (4.0%) | 0.239 |
| SGLT2 inhibitors | 1 (2.2%) | 1 (3.1%) | 3 (6.7%) | 2 (4.1%) | 0.811 |
| Thiazolidinediones | 0 (0.0%) | 0 (0.0%) | 0 (0.0%) | 1 (2.0%) | 0.999 |
| *Social determinants* | | | | | |
| Income (*n*, %) |  |  |  |  | 0.095 |
| < 1 MW | 13 (28.9%) | 5 (14.3%) | 10 (20.8%) | 7 (14.0%) |  |
| 1 a 3 MW | 30 (66.7%) | 24 (68.6%) | 35 (72.9%) | 38 (76.0%) |  |
| > 3 MW | 2 (4.4%) | 6 (17.1%) | 3 (6.2%) | 5 (10.0%) |  |
| Education level (*n,* %) |  |  |  |  | 0.074 |
| Low (primary education) | 18 (40.0%) | 12 (34.3%) | 21 (43.8%) | 23 (46.9%) |  |
| Medium (secondary education) | 22 (48.9%) | 21 (60.0%) | 27 (56.2%) | 19 (38.8%) |  |
| High (higher education) | 5 (11.1%) | 2 (5.7%) | 0 (0.0%) | 7 (14.3%) |  |
| Abbreviations: SIDD, Severe insulin-deficient diabetes; SIRD, Severe insulin-resistant diabetes; MOD, Mild obesity-related diabetes; MARD, Mild age-related diabetes; MW: Minimum Wages.  ^1^*p*-values calculated using Fisher’s exact test. | | | | | |
